# Attenuation of gadolinium enhancement in pituitary gland on magnetic resonance imaging of patients with pediatric growth hormone deficiency

**DOI:** 10.1186/s12880-023-01152-w

**Published:** 2023-11-17

**Authors:** Mariko Doai, Yuka Nishino, Yasuhiko Hayashi, Masatsune Ito, Munetaka Matoba

**Affiliations:** 1 Department of Radiology, Kanazawa Medical University, 1-1 Daigaku Uchinada Kahoku, Ishikawa, 920-0293 Japan; 2 Present Address: Department of Radiology, University of Toyama, 2630 Sugitani, Toyama, 930-0194 Japan; 3 Department of Neurosurgery, Kanazawa Medical University, 1-1 Daigaku Uchinada Kahoku, Ishikawa, Japan; 4 Department of Pediatrics, Kanazawa Medical University, 1-1 Daigaku Uchinada Kahoku, Ishikawa, Japan

**Keywords:** Growth hormone deficiency, Magnetic resonance imaging, Pituitary gland, Gadolinium enhancement

## Abstract

**Background:**

Although it is generally thought that disturbance of perfusion in the anterior lobe of the pituitary gland leads to complete or partial hypopituitarism, the gadolinium (Gd) enhancement findings on Magnetic Resonance Imaging (MRI) of patients with growth hormone deficiency (GHD) remain unknown. The purpose of this study was to compare Gd enhancement of the pituitary gland on MRI of patients with GHD to that of healthy subjects.

**Methods:**

In this retrospective study, we analyzed the data of 10 patients with clinically diagnosed GHD who underwent Gd-enhanced MRI of their pituitaries (age 8.3$\pm$3.5 year, female 1, males 9), together with data of 5 patients with clinically normal growth hormone (GH) dynamics who also underwent Gd-enhanced pituitary MRI (age 6.2$\pm$3.4 year, female 4, males 1). In each subject, a maximum-diameter region of interest (ROI) was drawn on the anterior pituitary gland of post Gd-enhanced coronal T1-weighted images, and the signal intensity ratio of the anterior pituitary gland to the white matter on the right temporal lobe of the same cross section was assessed.

**Results:**

The mean area of the ROI in the anterior pituitary gland and white matter of temporal lobe on the same cross section showed no significant differences between patients with GHD and those with normal GH (pituitary, 17.43 mm^2^$$\pm$$8.24 vs. 21.08 mm^2^$$\pm$$10.40, p = 1.00; white matter, 74.47mm^2^$$\pm$$24.19 and 62.50 mm^2^$$\pm$$17.90, p = 0.37), suggesting that the sizes of the pituitary glands were comparable. The ratios of Gd enhancement in the anterior pituitary gland showed significant differences between GHD and normal-GH subjects ($$\pm$$$$0.716\pm$$0.68$$\pm$$0.26 vs.$$0.72\pm$$0.16, p= 0.04).

**Conclusions:**

These results suggested that the contrast effect on Gd-enhanced MRI is attenuated in the pituitary glands of patients with GHD compared to those with normal GH. These new clinical findings regarding Gd-enhanced MRI can assist the diagnosis of pediatric GHD.

## Background

Growth hormone deficiency (GHD) is usually idiopathic, but some etiologies such as lesions causing GHD have been identified [1, 2]. Magnetic Resonance Imaging (MRI) with high tissue resolution is a standard method to detect pituitary lesions of patients with idiopathic-GHD (IGHD) [3, 4]. Although most IGHD patients don’t have pituitary lesions, previous studies have reported a small-size anterior lobe, a hypoplastic stalk, and an absent/ectopic bright spot of the posterior lobe as abnormalities in such patients [2, 5, 6].

At our hospital, we have noted a somewhat weak gadolinium (Gd)-contrast effect of the anterior lobe on MRI examination of IGHD patients. In those cases, it can be difficult to assess the pituitary gland for detection of potential lesions. The purpose of this study was to quantitatively compare the Gd contrast effect on MRI of the pituitary gland in patients with GHD.

## Methods

### Patients

This retrospective study was approved by the institutional review board, which waived the requirement for informed consent. Thirteen patients with GHD or suspected GHD underwent Gd-enhanced MRI at our hospital during the period from January 2016 to March 2020. We excluded patients whose provocation tests were normal, whose MRI and provocation tests were performed at different times, or who underwent MRI on a different system. Finally, 10 patients (age $\pm$8.3$\pm$3.5 year; female 1, male 9) with clinically diagnosed GHD who underwent Gd-enhanced pituitary MRI were enrolled. As control subjects, five patients with clinically normal GH function (age 6.2 ± 3.4 year; female 4, males 1) who underwent Gd-enhanced pituitary MRI were also enrolled as control healthy subjects.

All patients were short stature of -2 standard deviation (SD) or less according to the growth curve model.

To evaluate the severity of GHD in our patients, we used the classification system of the Study Group of Hypothalamo-Pituitary Disorder of the Ministry of Health, Labour and Welfare [7].

In this study, pediatric patients with GH peak levels of 3 ng/ml or below for insulin and arginine provocation tests were considered to have “severe” GHD. Otherwise, pediatric patients with peak GH levels of 6 ng/ml or below for insulin and arginine provocation tests were considered to have “moderate” disease, and those with GH peak levels of 6 ng/ml or less in two of three provocation tests: insulin, arginine, or clonidine were considered to have “mild” disease [7, 8].

### MRI protocol

We used a 3-T system (MAGNETOM Trio, A Tim system T-Class VB17, Siemens, Germany) to obtain T1-weighted fluid-attenuated inversion recovery (T1-FLAIR) and post-enhanced T1-FLAIR images after intravenous injection of contrast medium (0.1 mmol Gd/kg of body weight) using the following parameters: TR 2000 ms, TE 11 ms, TI 860 ms, FA 130, ETL 6, scan time 2 m 54 s, FOV 200 × 200 mm, slice thickness 2 mm, matrix size 320 × 320 and intersection gap 0.0 mm. For infants, the protocol differed slightly, with FOV reduction and 1.5-mm slice thickness. The imaging of post-enhanced T1-FLAIR images was performed immediately after injection of the contrast medium by manual pressure.

### Image analysis

**Fig. 1:**
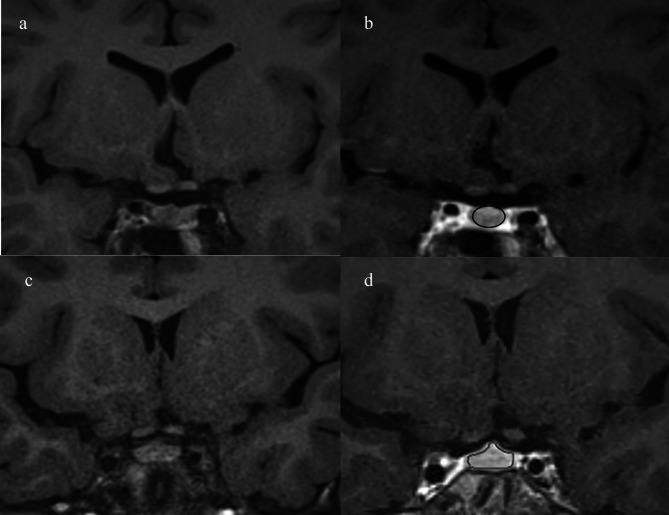
T1-FLAIR and Gd-enhanced MRI images of a 14-year-old male with severe GHD (**a**,**b**) and an 8-year-old male with normal GH function (**c**,**d**). The ROIs of the anterior pituitary gland were obtained at the maximum diameter in post-Gd-enhanced coronal T1-FLAIR images (arrow in b and d). The ratios of Gd-enhanced signals in the anterior pituitary gland to white-matter signals in the temporal lobe (signal intensity of the anterior pituitary gland / (signal intensity of the anterior pituitary gland + signal intensity of the right temporal lobe)) were 0.68 and 0.73, respectively

Two-dimensional regions of interest (ROI) in the anterior lobe of the pituitary gland were obtained at the maximum diameter in pre- and post-Gd enhanced coronal T1-FLAIR. Signal intensity ratios of the anterior lobe of the pituitary gland to the white matter on the right temporal lobe of the same cross section were calculated by the following formula: signal intensity of the anterior lobe of the pituitary gland / (signal intensity of the anterior lobe of the pituitary gland + signal intensity of the right temporal lobe) (Fig. 1).

The signal intensity of cerebral white matter is stable, and it can be traced an enough volume of the ROI, therefore, it was used as a relatively stable parameter as a signal ratio.

The ROI of the anterior pituitary gland was traced freehand according to its shape, and the ROI of the cerebral white matter on the right temporal lobe of the same cross section was traced in the largest plane using circular or oval ROI, all of the ROI were carefully traced to the interface to avoid contamination with the surrounding tissue.

The ROIs were measured by one radiologist (M.D.) with 16 years’ experience.

### Statistical analysis

Data are expressed as means ± SDs. The Mann-Whitney U test was used to compare average data of signal intensity ratios on post-Gd-contrast T1-FLAIR between the anterior lobe of the pituitary gland and the white matter in patients with GHD and that in patients with normal-GH-function. P values < 0.05 were considered significant. We tested interobserver variability using intraclass correlation coefficients (ICCs). Agreement was considered excellent if ICC > 0.8, good if 0.6‒0.8, moderate if 0.4‒0.6, and poor if < 0.4. All statistical calculations were done with PASW statistical software (ver. 23.0, SPSS, IBM, Chicago, IL).

## Results

We performed analysis on a total of 10 patients, of whom two had mild GH deficiency, one had moderate GH deficiency, and seven had severe GH deficiency (Table 1).

They ruled out panpituitary insufficiency by provocation tests, and familiality GHD ruled out by stature of patients and family history.

The interobserver analysis showed good to excellent agreement for the signal intensity ratios of the anterior pituitary gland to white matter of patients with GHD and those with normal GH function. The mean ICC of the signal intensity ratios of the anterior pituitary gland to white matter of patients with GHD was 0.981, and that of patients with normal GH function was 0.991. The mean ROI area in the anterior pituitary gland and white matter on the temporal lobe of the same cross section showed no significant differences between patients with GHD and those with normal GH function (pituitary, 17.43 mm^2^$$\pm$$8.24 and 21.08 mm^2^$$\pm$$10.4, p = 1.00; white matter, 74.47 mm^2^$$\pm$$24.19 and 62.50 mm^2^$$\pm$$17.90, p = 0.37, respectively) (Table 2). The ratios of Gd-enhancement in the anterior pituitary gland showed significant differences between GHD and normal GH ($$\pm$$$$0.716\pm$$0.68$$\pm$$0.26 vs.$$0.72\pm$$0.16, p= 0.04), while there no significant difference between GHD and normal GH patients in the ratios of unenhanced signals ($$\pm$$$$0.509\pm$$ 0.50$$\pm$$0.36 vs.$$0.51\pm$$0.15, p= 0.78) (Table 3).

**Table 1:** Patients and classification of GHD

Characteristics (n = 10)	Values
Age (yrs)	
Mean (± SD)	8.3 $\pm$3.5 year, female 1, males 9
range	3–14
SD of stature by growth curve model	-2.60 $\pm$-0.69
range	-4.42 - -2.00
GHD severity	
mild	2
moderate	1
severe	7

**Table 2:** The mean area ROI in the anterior pituitary gland and temporal lobe white matter of GHD patients vs. normal-GH-function pediatric patients (Mann-Whitney U test)

Type	GHD	Normal GHD	p-value
APG mm^2^	17.43$\pm$8.24	21.08$\pm$10.40	1.00
WM mm^2^	74.47$\pm$24.19	62.50$\pm$17.90	0.37

Data are means ± SDs, APG, anterior pituitary gland; WM, white matter of temporal lobe

**Table 3:** The ratios of pre-enhancement and Gd-enhanced signals in the anterior pituitary gland of GHD patients vs. normal-GH-function pediatric patients (Mann-Whitney U test)

Type	GHD	Normal GHD	p-value
Pre APG/APG + WM	0.50$\pm$0.36	$0.51\pm$0.15	0.78
Post Gd APG/APG + WM	0.68$\pm$0.26	$0.72\pm$0.16	0.04*

Data are means ± SDs, * *p*<0.05

## Discussion

Our study compared the differences in Gd-enhancement of the anterior pituitary gland in patients with GHD and those with normal GH function on MRI. The signal ratios to be compared were set to the white matter of the temporal lobe at the same level as the maximum surface of the anterior pituitary gland where signals were stable, and ROI volumes in 2D were obtained. The results showed that the Gd-enhancement ratios between the anterior pituitary gland and the white matter of the temporal lobe were significantly lower in GHD patients than in pediatric subjects with normal GH function. Previously, Wang et al. reported that IGHD and the degree of GHD are associated with non-regional perfusion delay in morphologically normal adenohypophyses [1]. They suggested that lack of lateralization of perfusion delay was due to structural abnormalities in the microvasculature of IGHD [1]. Furthermore, Maghnie et al. evaluated pituitary vascularization in children with hypopituitarism, central diabetes, or Langerhans cell histiocytosis using dynamic MR images. They reported that isolated GHD patients had limited vascular damage, and that isolated GHD patients with perinatal hypoxemia and diabetes insipidus may develop progressive pituitary atrophy and multiple pituitary hormone deficiency due to chronic pituitary hypoperfusion [9].

Although our study evaluated only post-Gd-enhancement images, we suspect that patients with GHD have at least slight blood flow reduction in their anterior pituitary gland. It is assumed that this result is caused by abnormal development of the microvasculature in GHD patients, as reported by Wang et al. If blood flow is shown to be inversely related with severity of GHD, attenuation of Gd-enhancement of the pituitary gland in GHD would be an effective evaluation method for diagnosis and monitoring of treatment effect. In addition, it has been elucidated using with immunochemistry that somatotrophs are abundant in the lateral and posterolateral portions of the anterior pituitary gland, but Wang et al. found no significant difference in regional perfusions in 10 ROIs of the anterior pituitary gland [1]. Liu et al. evaluated the time-enhancement curves of selected regions in the anterior pituitary gland by dynamic enhancement MRI in patients with pituitary dwarfism and normal pituitary function and found that the time to achieve maximum enhancement was delayed in pituitary dwarfism [10].

Considering these results, it is assumed to be useful only to evaluate the speed of attenuation of the Gd-contrast effect on MRI at the pituitary gland in GHD. There are some study limitations to consider: (1) Our study population was very small. It is necessary to increase the number of cases in the future. In addition, very few control subjects with normal GH underwent contrast-enhanced brain MRI under the same conditions as those with GHD. (2) We did not evaluate between-patient differences due to differing severity of GHD or within-patient changes due to therapeutic effects. (3) This study evaluated only post-Gd-contrast images and did not evaluate blood flow using dynamic contrast or perfusion images. Quantitative and semi-quantitative evaluations using perfusion images and dynamic contrast are required in the future. In addition, it will be necessary to examine the relationships between these quantitative or semi-quantitative evaluations and the post-contrast ratios determined in this study.

## Conclusions

The Gd-contrast effect on MRI of the pituitary gland is attenuated in IGHD patients compared to those with normal GH function. These new clinical findings regarding Gd-enhanced MRI can assist the diagnosis of pediatric GHD.

## Data Availability

The datasets used and / or analyzed during the current study are available from the corresponding author on reasonable request.

## Abbreviations

2D: 2-dimensions
Gd: Gadolinium
GH: Growth hormone
GHD: Growth hormone deficiency
ICCs: Intraclass correlation coefficients
IGHD: Idiopathic growth hormone deficiency
MRI: Magnetic Resonance Imaging
ROI: Region of interest
SD: Standard deviation
T1-FLAIR: T1-weighted fluid-attenuated inversion recovery
